# Impact of Rheumatoid Arthritis Disease Activity Test on Clinical Practice

**DOI:** 10.1371/journal.pone.0063215

**Published:** 2013-05-07

**Authors:** John W. Peabody, Vibeke Strand, Riti Shimkhada, Rachel Lee, David Chernoff

**Affiliations:** 1 Departments of Epidemiology & Biostatistics and Medicine, University of California San Francisco, San Francisco, California, United States of America; 2 QURE Healthcare, San Rafael, California, United States of America; 3 Division of Immunology/Rheumatology, Stanford University, Stanford, California, United States of America; 4 Department of Health Policy and Management, University of California Los Angeles, Los Angeles, California, United States of America; 5 Crescendo Bioscience, South San Francisco, California, United States of America; University Hospital Jena, Germany

## Abstract

**Background:**

Variability exists in the assessment of disease activity in rheumatoid arthritis (RA) patients that may affect quality of care.

**Objectives:**

To measure the impact on quality of care of a Multi-Biomarker Disease Activity (MBDA) test that quantitatively assesses RA disease activity.

**Methods:**

Board-certified rheumatologists without prior experience with the MBDA test (N = 81) were randomized into an intervention or control group as part of a longitudinal randomized-control study. All physicians were asked to care for three simulated RA patients, using Clinical Performance and Value (CPV™) vignettes, in a before and after design. CPV™ vignettes have been validated to assess the quality of clinical practice and identify variation in care. The vignettes covered all domains of a regular patient visit; scores were determined as a percentage of explicit predefined criteria completed. Three vignettes, representing typical RA cases, were administered each round. In the first round, no physician received information about the MBDA test. In the second round, only physicians in the intervention group were given educational materials about the test and hypothetical test results for each of the simulated patients. The outcome measures were the overall quality of care, disease assessment and treatment.

**Results:**

The overall quality scores in the intervention group improved by 3 percent (p = 0.02) post-intervention compared with baseline, versus no change in the control group. The greatest benefit in the intervention group was to the quality of disease activity assessment and treatment decisions, which improved by 12 percent (p<0.01) compared with no significant change in the control group. The intervention was associated with more appropriate use of biologic and/or combination DMARDs in the co-morbidity case type (p<0.01).

**Conclusions:**

Based on these results, use of the MBDA test improved the assessment and treatment decisions for simulated cases of RA and may prove useful for rheumatologists in clinical practice.

## Introduction

Rheumatoid arthritis (RA) is a chronic, systemic inflammatory disorder that affects approximately 1.5 million people in the United States.[1] Unlike disease areas such as cardiology that have extensive research and appropriate use criteria, evidence-based medicine for RA diagnosis, evaluation and treatment are based on largely subjective criteria.[2], [3] Until 2010, the classification criteria for RA had not changed in 23 years.[4] Recent treatment guidelines direct rheumatologists to treat RA promptly and aggressively, aiming for remission as a therapeutic target.[5], [6] Nevertheless, early recognition of signs and symptoms and timely referral to rheumatologists remain challenging; appropriate use of non-biologic and biologic disease-modifying anti-rheumatic drugs (DMARDs) significantly improves outcomes but requires close monitoring of disease activity, including laboratory evaluations and imaging that is often difficult to achieve.[3], [7] A number of composite tools are available for assessing disease activity in RA, of which 6 have been recommended by the American College of Rheumatology (ACR).[8] They are infrequently used in practice and have limitations, including reliance on subjective measures with frequent discordance between physician and patient assessments.[9] Patient global assessment and its contribution to the ACR/European League Against Rheumatism remission criteria are affected by non-inflammatory factors.[10] Moreover, individual variability in therapeutic responses and lack of objective biomarkers for identification of active disease can lead to variability in patient care that may affect the management of RA [11]–[15].

New technology and diagnostic tools have been shown to lower variability in clinical areas such as cardiology, radiology and oncology, [16] but so far this effect has not been demonstrated in rheumatology. Reducing the variability in rheumatologic assessments and treatment decisions would be beneficial in RA not only because it would lead to higher quality for the patient but it could lower aggregate health care costs. [17], [18] By objectively quantifying disease activity in RA, physicians may be better equipped to allocate resources accurately, avoid ordering unnecessary tests, and change therapy effectively [19].

A multi-biomarker disease activity (MBDA) blood test (Vectra DA™; Crescendo Bioscience, Inc., South San Francisco, CA) is currently available in the U.S. to assess disease activity in adult patients with RA. The test is done by taking serum prepared from patient peripheral blood and sending it to a central laboratory for automated, multiplexed measurement of 12 protein biomarkers that play key roles in the underlying pathophysiology of RA. The MBDA test is based on an algorithm that uses the concentrations of the 12 biomarkers to generate a score that represents the level of RA disease activity on a scale of 1 (lowest activity) to 100 (greatest)[20], [21] and, in studies of analytical validation, the test is precise and reproducible.[20] The MBDA test was developed to correlate with the 28-joint Disease Activity Score (DAS28) and has been clinically validated by correlations with DAS28 and other disease activity measures in independent RA cohorts, with thresholds established for low, moderate and high disease activity.[22], [23] Other studies show that the MBDA test tracks responses to treatment with biologic and non-biologic DMARDs,[22], [23] and may potentially be an indicator of progressive joint damage in patients with RA.[24] The MBDA test is not validated for diagnosing RA.

There are currently no blood tests that are specific for RA. The two clinical laboratory tests most closely associated with RA, rheumatoid factor and anti-citrullinated protein antibody tests, are for diagnosis and prognosis. The only blood tests that are routinely used to assess disease activity in RA are the erythrocyte sedimentation rate (ESR) and C-reactive protein (CRP). Both have limitations, as their values are in the normal range in up to half of patients with active disease, and they are often discordant with each other.[25], [26] Roles for the MBDA test in RA patient management include situations where clinical evaluations and laboratory test results provide a mixed picture of disease activity, or assessment is confounded by symptoms from diseases other than RA. The MBDA test is well suited to being studied with case simulations because, as an objective test, the inclusion of a hypothetical MBDA score in a case simulation is sufficient to duplicate its real-life inclusion in the clinical decision-making process. Thus to evaluate the potential clinical utility of the MBDA test, we used simulated cases of RA patients and hypothetical MBDA scores to evaluate clinical practice by rheumatologists before and after exposure to the MBDA test.

## Methods

### Overview

This is a longitudinal randomized controlled study of practicing rheumatologists from across the U.S. with no prior experience with the MBDA test. In a “before and after” design, rheumatologists were asked to care for simulated patients with RA, using Clinical Performance and Value (CPV™) vignettes via web-based interactive sessions. Physicians completed the vignettes at baseline (3 cases pre-intervention, Round 1) and again 12 weeks later (3 cases post-intervention, Round 2). In Round 1, none of the rheumatologists received any information about the MBDA test and none of the vignettes included MBDA scores. In Round 2, rheumatologists who were randomized to the intervention group were introduced to the MBDA test and provided with a hypothetical test result for each Round 2 vignette, whereas MBDA test information and results were not made available to physicians randomized to the control group. This study was conducted in accordance with the ethical standards of the institutional review board (IRB), approved by the Essex IRB, Lebanon, NJ. Participants provided written consent to participate, and Essex IRB approved the consent form.

### The Intervention

The intervention group received hypothetical MBDA scores, on a scale from 1 to 100, for each patient in the Round 2 vignettes. As is true for the actual MBDA test, lower scores indicated less disease activity and higher scores generally indicated a need to initiate or change therapy. Prior to receiving hypothetical MBDA scores rheumatologists in the intervention group were provided with information about the MBDA test and offered a variety of educational modalities, including emailed materials, mailed materials, access to a podcast, invitation to a live webinar and/or access to the Principal Investigator of the study and the Chief Medical Officer of Crescendo Bioscience for answers to specific questions.

### Eligibility and Selection of Physicians

**Figure 1:**
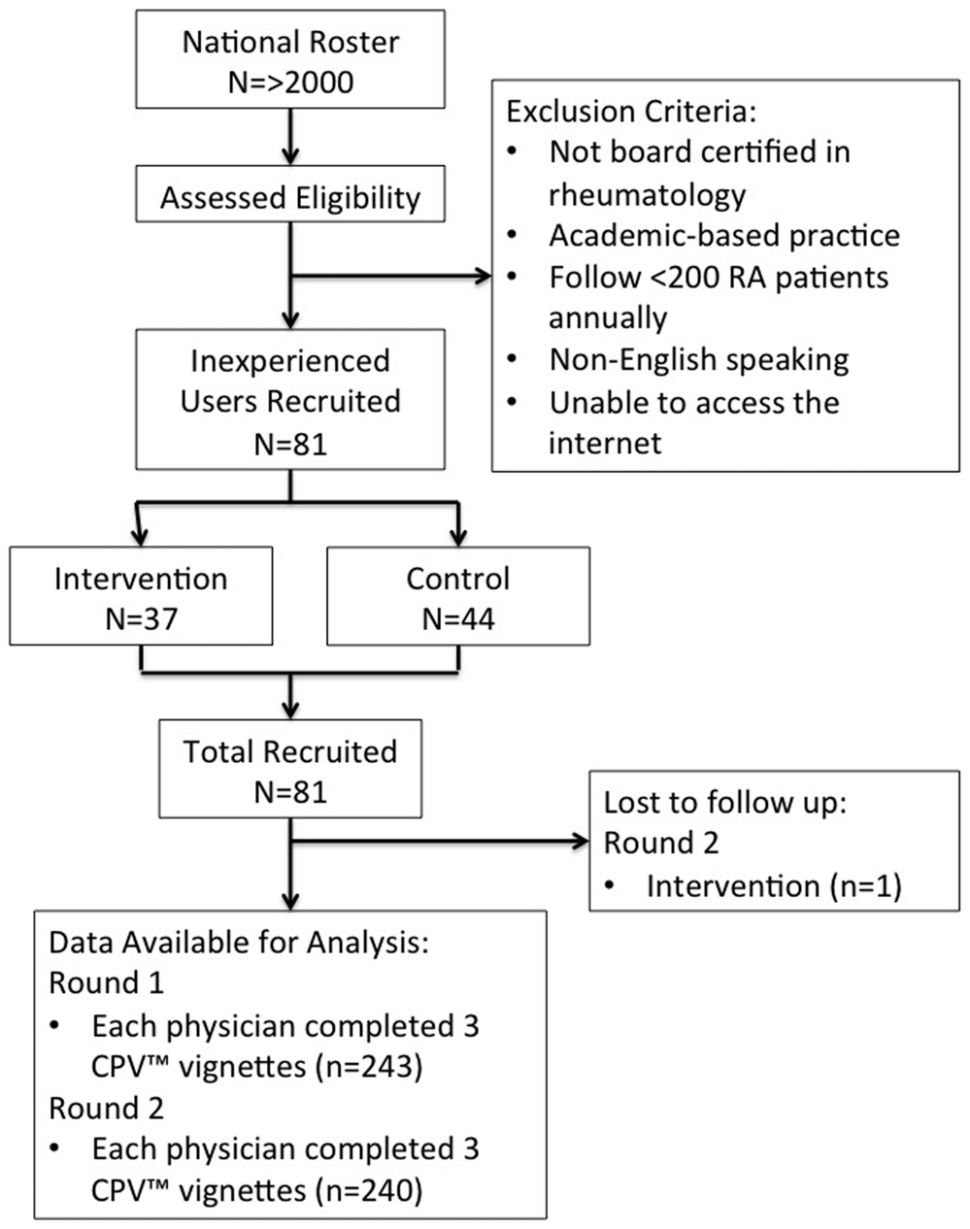
Flow diagram of sample selection.

Eligibility for participation in the study was assessed using a physician questionnaire. Physicians had to be (1) board-certified rheumatologists, (2) English-speaking, (3) practicing in a community/non-academic based setting, (4) in practice with >200 RA patients in their care, (5) accessible by Internet, and (6) inexperienced with the MBDA test. From a nationally representative list of 2600 eligible rheumatologists, 81 physicians were selected for participation in this study (Figure 1). These physicians were randomized into one of two arms: 37 in the intervention arm, and 44 in the control arm. Only one physician was lost to follow-up between baseline and post-intervention assessments.

### Clinical Performance and Value™ vignettes

The quality of clinical performance was measured at baseline (Round 1) and after 12-weeks (Round 2) using Clinical Performance and Value (CPV™) vignettes. CPV™ vignettes have been demonstrated to be a validated means for assessing differences in clinical practice and inherent variation in care independent of case mix.[27]–[29] The vignettes used in this study simulated clinical encounters involving patients with RA. In each vignette, the rheumatologists provided open-ended responses regarding clinical care. Responses were scored in 5 domains: taking a medical history, performing a physical examination, ordering appropriate laboratory and imaging tests, assessing disease activity and physical function, and prescribing treatment. Scoring criteria were derived from a literature review and expert opinion to generate an explicit evidence base. Scores for the completed vignettes were the percentage of physician answers that matched these evidence-based criteria.

Three types of CPV™ vignettes, representing typical cases seen by practicing rheumatologists, were prepared to evaluate the impact of the MBDA test result on patient management. Four vignettes were prepared for each type. Type A vignettes comprised RA cases insufficiently controlled with treatment and requiring addition of or increase in dosage of a non-biologic DMARD; Type B vignettes included RA cases who received inadequate treatment judged to require addition of a non-biologic or biologic DMARD; and Type C vignettes described cases of adequate treatment for RA but worsening symptoms due to one of four co-morbidities (depression, fibromyalgia, adenocarcinoma metastatic to bone and osteoarthritis). All physicians completed 2 randomly assigned cases for each of the 3 case types, one at baseline and one 12 weeks later or after the intervention, for a total of 6 vignettes per physician. Physicians were surveyed at the end of each round after completing 3 cases and asked what percentage of their cases the vignettes represented. On average the 3 case types in this study represented 62% to 80% of the types of patients that the rheumatologists in this study saw in their practices.

### Analysis

Our primary hypothesis was that the MBDA test would improve quality of care. Quality was measured by the CPV™ vignettes. We used the overall quality score and in subsequent analyses determined that the disease activity assessment and treatment quality domain scores were the domains most affected by introduction of the MBDA test, as might be expected. As a corollary, we further hypothesized that quality effects would occur across all vignette case types (Types A, B, and C). Secondary outcomes included appropriate use of non-biologic or biologic DMARDs or combinations, number of imaging tests ordered, number of laboratory tests ordered, and use of other resources (which included use of office visits).

We used multivariate regression modeling to compare outcomes in Round 2 versus Round 1 in the intervention and the control groups. For the primary outcomes ― overall CPV™ quality scores and scores for the combined disease assessment and treatment domains–we used linear regression models of continuous data. Regression coefficients in the model included dummy variables for (1) intervention versus control groups, which captured whether the intervention group was significantly different from controls at baseline, (2) intervention group scores at Round 2 versus baseline, which captured changes over time in the intervention group, (3) control scores at Round 2 versus baseline, which captured changes over time in the control group, and (4) physician characteristics identified *a priori* as potential confounders: age, gender, years of rheumatology practice, number of days worked per week, practice location, single or multispecialty practice, employed by a health system, use/employ mid-level practitioners in office, on-site phlebotomy, number of patients seen per week and number of RA patients, and insurance coverage of patients (percent under Medicaid and percent under Medicare). We also included the interaction between facility variables: mid-level practitioners and multi-specialty facility.

For the secondary outcome, appropriate use of biologic or combination DMARDs, we used logistic regression controlling for the same variables as described above. We used a Poisson distribution for the number of imaging tests ordered, number of laboratory tests ordered, and use of resources. Regression models of the secondary outcomes controlled for the same co-variates described above for the primary outcomes. All analyses were conducted using STATA 12.0.

## Results

There were no significant differences in physician characteristics between the intervention and control groups at baseline, with the exception of a difference in mid-level providers at the facility (Table 1). Quality scores, measured by CPV™ overall and disease activity assessment and treatment domain scores, differed significantly at baseline between the three case types, which reflected the different clinical scenarios completed by rheumatologists (Table 2). Type C cases, which presented patients with potentially confounding co-morbidities, resulted in the lowest baseline scores.

**Table 1:** Baseline Characteristics of Intervention and Control group physicians, All Case types.

	Intervention (n = 37)	Control (n = 44)	p-value
Number of rheumatology cases seen ≥30 per week	48.65	62.79	0.21
% of Medicare patients	40.03	38.63	0.61
% of Medicaid patients	7.86	7.51	0.88
On-site Phlebotomy	78.38	74.42	0.68
Employed by a system	21.62	20.93	0.94
Male	75.68	67.44	0.42
Practice at least 5/days per week	70.27	58.14	0.27
Age	49.70	48.95	0.74
Facility is multi-specialty	48.65	55.81	0.53
Facility has midlevel providers	35.14	6.98	<0.001

**Table 2:** Baseline Mean CPV Overall and Disease Assessment and Treatment Quality Scores for All Study Physicians.

	Mean	Standard deviation	p-value
			(Relative to Type C)
Overall CPV Score			
All Types	60.36	12.11	n/a
CPV Type A	61.97	12.48	<0.001
CPV Type B	62.01	11.69	<0.001
CPV Type C	57.09	11.58	n/a
CPV Disease Assessment and Treatment Domain Score			
All Types	54.01	21.71	n/a
CPV Type A	61.73	21.98	<0.001
CPV Type B	53.25	20.88	0.03
CPV Type C	47.04	19.86	n/a

CPV indicates Clinical Performance and Value.

When we evaluated the impact of introducing the MBDA test into clinical practice on disease assessment and treatment quality scores, rheumatologists in the intervention group improved their quality scores by 12 percentage points for the 3 case types combined, after adjustment for the other covariates (p<0.001). This improvement was also significant for each of the 3 case types individually. The regression results, using combined CPV™ disease activity assessment and treatment domain scores, for all cases and disaggregated, are shown in Table 3. As expected, in the control group, no statistically significant improvements in treatment decisions were evident between Rounds 1 and 2.

**Table 3:** Multiple Linear Regression Models of CPV Disease Assessment and Treatment Scores by Case Type for All Cases and for Each Case Type.

	All Case Types		Type A		Type B		Type C	
	Coef	p-value	Coef	p-value	coef	p-value	coef	p-value
MBDA test Intervention at Round 2 compared with Baseline	12.49	<0.001	13.06	0.01	11.56	0.02	14.23	<0.001
Control at Round 2 compared with Baseline	3.02	0.24	0.06	0.99	1.55	0.73	7.64	0.08
MBDA test Intervention compared with control at Baseline	−4.68	0.09	−6.24	0.22	−6.15	0.22	−1.87	0.70
Number of rheum cases seen ≥30 per week	1.55	0.48	2.77	0.49	−0.03	0.99	2.79	0.47
% of Medicare patients	0.04	0.67	0.02	0.89	0.16	0.30	−0.09	0.55
% of Medicaid patients	−0.23	0.03	−0.35	0.08	−0.14	0.45	−0.30	0.11
On-site Phlebotomy	−5.19	0.03	−4.16	0.33	−9.19	0.03	−3.05	0.46
Employed by a system	7.82	<0.001	12.66	<0.001	2.53	0.54	7.43	0.07
Male	0.62	0.79	0.56	0.89	4.00	0.32	−1.87	0.64
Practice at least 5 days per week	−1.05	0.62	−1.22	0.75	−3.01	0.42	1.71	0.65
Age	−0.16	0.14	−0.27	0.17	−0.04	0.86	−0.12	0.52
Facility is multi-specialty	3.37	0.13	5.81	0.15	0.21	0.96	4.54	0.25
Facility has midlevel providers	0.36	0.93	8.10	0.29	−1.89	0.81	−5.51	0.46
Facility has midlevel providers and is multi-specialty	−6.65	0.20	−12.07	0.20	−3.37	0.72	−3.65	0.69
Constant	66.05	<0.001	76.32	<0.001	60.37	<0.001	58.94	<0.001

CPV indicates Clinical Performance and Value; MBDA, multi-biomarker disease activity.

When we looked at the impact on the overall quality scores, there was also a statistically significant improvement in the intervention group compared with controls following use of the MBDA test (Table 4). Physicians in the intervention group improved their scores by 3 percentage points overall in the 3 case types combined, after adjustment for the other covariates. These effects did not differ by case type (regression analysis results not shown). In the control group there were no significant improvements between Rounds 1 and 2.

**Table 4:** Multiple Linear Regression Model of Overall CPV scores for all Case Types.

	Coef	p-value
MBDA test Intervention at Round 2 compared to Baseline	3.51	0.02
Control at Round 2 compared to Baseline	1.97	0.17
MBDA test Intervention compared to control at Baseline	−0.36	0.82
Number of rheum cases seen> = 30 per week	1.06	0.40
% of Medicare patients	−0.05	0.31
% of Medicaid patients	−0.26	<0.001
On-site Phlebotomy	−3.65	0.01
Employed by a system	4.39	<0.001
Male	−0.17	0.89
Practice at least 5/days per week	−1.97	0.10
Age	−.0001	.999
Facility is multi-specialty	3.36	0.01
Facility has midlevel providers	−0.84	0.73
Facility has midlevel providers and is multi-specialty	−5.91	0.05
Constant	66.57	<0.001

CPV indicates Clinical Performance and Value; MBDA, multi-biomarker disease activity.

Analysis of covariates controlled for in the quality models revealed some associations: employment in a health system resulted in a score that was greater by 8 percentage points for the combined disease activity assessment and treatment domain, and by 4 percentage points for the overall score. Analysis of overall scores revealed they decreased slightly (by 0.26 percentage point) with each percent increase in the portion of Medicaid patients in a practice. Outcome scores for rheumatologists employed by a health care system and for those working in multi-specialty practices were significantly greater, by 4 and 3 percentage points respectively. For disease activity assessment and treatment quality outcomes, the effect of working in a multi-specialty practice was similar (3 percentage points) and an even greater effect (8 percentage points) was observed for rheumatologists working in a health care system (Table 3).

Analysis of the secondary endpoints revealed that in the intervention group there was significant improvement in the appropriate use of biologic or combination DMARDs in patients with co-morbidities; however, this association was only evident in case Type C, for which no change in RA therapy was appropriate, and not the others (p = 0.008). The significant marginal effects for the MBDA test here indicated that rheumatologists in the intervention group were 10 percentage points more likely to appropriately use biologic or combination DMARDS compared with baseline. There was no significant change in the likelihood of correctly using biologics in the other two case types or in the control group over time. There was also no observable impact of use of the MBDA test in the models examining imaging, laboratory use, and referrals (combined resource use), either for case types combined or individually.

## Discussion

This study examined the impact of introducing an MBDA test on the quality of clinical practice for simulated patients with RA. Findings indicate that having knowledge of and access to the MBDA test result translated into better assessment and treatment decision-making by rheumatologists, evidenced by improved patient vignette scores in the intervention group compared with controls. These results, observed in a randomized controlled design, support the findings of earlier studies indicating that offering physicians a quantitative measure of disease activity improves their practice and may lead to better clinical outcomes.[19], [30] In RA in particular, the lack of more objective measures of disease activity can make it difficult for physicians to make the most appropriate treatment decisions. The effect of the MBDA test intervention here indicates that by using a fully objective indicator of disease activity, rheumatologists may be able to more effectively assess and treat a variety of typical RA cases.

Poor quality and clinical variation are a challenge in general practice and specialty care.[31] New technology, however, is often not considered in discussions about how to improve practice where, increasingly, the health care conversation has turned to new payment models, performance incentives and care reorganization. However, advances in technology that provide physicians with information that cannot be derived from the physical exam or from a non-specific laboratory test, such as ESR or CRP, may help with decisions of whether to maintain or alter treatment. In this study the MBDA test led to significant improvement in quality for the combined domain of clinical assessment and treatment, where benefit is the most plausible, compared with the other domains that were evaluated–taking a medical history, performing a physical examination, and ordering appropriate laboratory and imaging tests. Thus, introduction of the MBDA test helped eliminate clinical and therapeutic ambiguity, resulted in better treatment decisions and improved quality of care for the RA cases represented in the vignettes.

These results further indicate that the MBDA test was particularly useful in those cases where there was an underlying co-morbidity, evident by the significant estimate of effects of the intervention on disease assessment and treatment domain quality scores in the type C vignettes where there was significant improvement in the appropriate use of biologic or combination DMARDs. The MBDA test thus appears to have helped rheumatologists distinguish between symptoms of RA and a co-morbid condition, and thereby avoid changing RA therapy when the new complaint was due to a different disease.

There are limitations to this study. First, our sample size was relatively small and thus likely limited our statistical power for the secondary outcomes. Second, because the control group received neither MBDA training, nor MBDA test results, the study did not directly address the independent effect of training alone. It seems likely, however, that little or no effect should have resulted from training independent of the MBDA test result. The study did not analyze the long-term impact of use of the MBDA test on physician practice and resulting improvements in patient outcomes; a third round of vignette administration could demonstrate whether the improvements observed were sustained beyond the limited study period. In addition, the MBDA test potentially may be used for long-term disease tracking, and this assessment of its use at a single time point, may not fully assess its impact on routine practice. Future studies could be designed to overcome these limitations by using a larger sample and longer time frame. Future work might also include additional case types, patient outcomes linked to the two groups of rheumatologists, sampling of academic practices and more analysis on resource utilization. Importantly, although the MBDA test results used in this study were designed to represent a real-world test, these results were hypothetical and might differ from those obtained for actual patients.

The methodology used in this study may prove particularly helpful to others wanting to demonstrate clinical effectiveness. Traditional clinical effectiveness trials are costly, confounded by case mix variation among different sites, and typically exceed the budgets of entrepreneurs, potentially stifling innovation. [32] By comparison, we used six CPV™ vignettes, expressly written for this study in RA, representing three of the most common types of RA cases: patients inadequately treated with non-biologic DMARDs, patients inadequately treated with a non-biologic DMARD in combination with another non-biologic or a biologic DMARD, and patients with co-morbidities. The case simulations used in this study have been rigorously validated against standardized patients in two large studies and shown to match actual practice.[28], [29] Moreover, research using this evaluation technology has shown that higher quality vignette scores reflect better patient care.[27], [33], [34]

Expanding physician knowledge about critical details of the patient's disease not only facilitates clinical decision-making but may also optimize resource allocation by matching the best treatment to the individual patient type. It is not clear what impact this will have on costs. In RA, better precision in assessing disease activity may result in more appropriate use of non-biologic and/or biologic DMARDs, thereby reducing cost and risk of significant adverse effects that may be associated with biologic DMARDs. In other cases, better precision in assessing disease activity may indicate an earlier need for biologic DMARDs, which in turn can improve long-term outcomes, including lowering costs.

In spite of the limitations, this study shows that the availability of an MBDA test in clinical practice may impact decision-making and improve quality of care for patients with RA. Novel technologies for assisting physicians to assess RA disease activity and more appropriately treat patients should be similarly evaluated for gauging the impact on clinical care.

## References

[1] Sacks JJ, Luo YH, Helmick CG (2010) Prevalence of specific types of arthritis and other rheumatic conditions in the ambulatory health care system in the United States, 2001-2005. Arthritis Care Res (Hoboken) 62: 460–4. 20391499 10.1002/acr.20041

[2] Fuster V, Ryden LE, Asinger RW, Cannom DS, Crijns HJ, et al (2001) ACC/AHA/ESC Guidelines for the Management of Patients With Atrial Fibrillation: Executive Summary A Report of the American College of Cardiology/American Heart Association Task Force on Practice Guidelines and the European Society of Cardiology Committee for Practice Guidelines and Policy Conferences (Committee to Develop Guidelines for the Management of Patients With Atrial Fibrillation) Developed in Collaboration With the North American Society of Pacing and Electrophysiology. Circulation 104: 2118–50. 11673357

[3] Majithia V, Geraci SA (2007) Rheumatoid arthritis: diagnosis and management. Am J Med 120: 936–9. 17976416 10.1016/j.amjmed.2007.04.005

[4] Aletaha D, Neogi T, Silman AJ, Funovits J, Felson DT, et al (2010) Rheumatoid arthritis classification criteria: an American College of Rheumatology/European League Against Rheumatism collaborative initiative. Arthritis Rheum 62: 2569–81. 20872595 10.1002/art.27584

[5] Smolen JS, Aletaha D, Bijlsma JW, Breedveld FC, Boumpas D, et al (2010) Treating rheumatoid arthritis to target: recommendations of an international task force. Ann Rheum Dis 69: 631–637. 20215140 10.1136/ard.2009.123919 PMC3015099

[6] Smolen JS, Landewe R, Breedveld FC, Dougados M, Gaujoux-Viala C, et al (2010) EULAR recommendations for the management of rheumatoid arthritis with synthetic and biological disease-modifying antirheumatic drugs. Ann Rheum Dis 69: 964–75. 20444750 10.1136/ard.2009.126532 PMC2935329

[7] Singh JA, Furst DE, Bharat A, Curtis JR, Kavanaugh AF, et al (2012) Update of the 2008 American College of Rheumatology recommendations for the use of disease-modifying antirheumatic drugs and biologic agents in the treatment of rheumatoid arthritis. Arthritis Care Res 64: 625–639. 10.1002/acr.21641 PMC4081542 22473917

[8] Anderson J, Caplan L, Yazdany J, Robbins ML, Neogi T, et al (2012) Rheumatoid arthritis disease activity measures: American College of Rheumatology recommendations for use in clinical practice. Arthritis Care Res 64: 640–7. 10.1002/acr.21649 PMC4028066 22473918

[9] Studenic P, Rader H, Smolen JS, Aletaha D (2012) Arthritis Rheum Discrepancies between patients and physicians in their perceptions of rheumatoid arthritis disease activity. Arthritis Rheum 64: 2814–23. 22810704 10.1002/art.34543

[10] Masri KR, Shaver KS, Shadouri SH (2012) Validity and reliability problems with patient global as a component of the ACR/EULAR remission criteria as used in clinical practice. J Rheumatol (39): 1139–45. 10.3899/jrheum.111543 22589262

[11] Beresniak A, Russell AS, Haraoui B, Bessette L, Bombardier C, et al (2007) Advantages and limitations of utility assessment methods in rheumatoid arthritis. J Rheumatol 34: 2193–200. 17937471

[12] Bykerk VP, Schieir O, Akhavan P, Hazlewood GS, Cheng CK, et al (2011) Emerging Issues in Pharmacological Management of Rheumatoid Arthritis: Results of a National Needs Assessment Survey Identifying Practice Variations for the Development of Canadian Rheumatology Association Clinical Practice Recommendations. J Rheumatol 39: 1555–8. 21885494 10.3899/jrheum.110208

[13] Lacaille D, Anis AH, Guh DP, Esdaile JM (2005) Gaps in care for rheumatoid arthritis: a population study. Arthritis Rheum 53: 241–8. 15818655 10.1002/art.21077

[14] MacLean CH, Louie R, Leake B, McCaffrey DF, Paulus HE, et al (2000) Quality of care for patients with rheumatoid arthritis. JAMA 284: 984–92. 10944644 10.1001/jama.284.8.984

[15] Peabody JW, Strand V, Chernoff D, Ta HM (2012) Evaluation of Clinical Performance and Variation Among Three Types of Patients with Rheumatoid Arthritis: Opportunities to Raise Quality and Lower Costs. ISPOR 17th Annual International Meeting; Washington D.C.

[16] Jiang Y, Nishikawa RM, Schmidt RA, Toledano AY, Doi K (2001) Potential of computer-aided diagnosis to reduce variability in radiologists' interpretations of mammograms depicting microcalcifications. Radiology 220: 787–94. 11526283 10.1148/radiol.220001257

[17] Butcher L (2009) Health plans and providers struggle to fix payment system. Manag Care 18: 30–3. 19472565

[18] Panella M, Marchisio S, Di Stanislao F (2003) Reducing clinical variations with clinical pathways: do pathways work? Int J Qual Health Care 15: 509–21. 14660534 10.1093/intqhc/mzg057

[19] Farheen K, Agarwal SK (2011) Assessment of disease activity and treatment outcomes in rheumatoid arthritis. J Manag Care Pharm 17: S09–13. 22073934 10.18553/jmcp.2011.17.s9-b.S09 PMC10438217

[20] Eastman PS, Manning WC, Qureshi F, Haney D, Cavet G, et al (2012) Characterization of a multiplex, 12-biomarker test for rheumatoid arthritis. J Pharm Biomed Anal 70: 415–424. 22749821 10.1016/j.jpba.2012.06.003

[21] Zhao X, Qureshi F, Eastman PS, Manning WC, Alexander C, et al (2012) Pre-analytical effects of blood sampling and handling in quantitative immunoassays for rheumatoid arthritis. J Immunol Methods 30: 72–80. 10.1016/j.jim.2012.02.007 PMC3404505 22366959

[22] Bakker MF, Cavet G, Jacobs JW, Bijlsma JW, Haney DJ, et al (2012) Performance of a multi-biomarker score measuring rheumatoid arthritis disease activity in the CAMERA tight control study. Ann Rheum Dis 71: 1692–1697. 22596166 10.1136/annrheumdis-2011-200963 PMC3439649

[23] Curtis JR, van der Helm-van Mil AH, Knevel R, Huizinga TW, Haney DJ, et al (2012) Validation of a novel multi-biomarker test to assess rheumatoid arthritis disease activity. Arthritis Care Res 64: 1794–803. 10.1002/acr.21767 PMC3508159 22736476

[24] van der Helm-van Mil AH, Knevel R, Cavet G, Huizinga TW, Haney DJ (2012) An evaluation of molecular and clinical remission in rheumatoid arthritis by assessing radiographic progression. Rheumatology: in press. 10.1093/rheumatology/kes378 PMC3630394 23287359

[25] Graf J, Scherzer R, Grunfeld C, Imboden J (2009) Levels of C-reactive protein associated with high and very high cardiovascular risk are prevalent in patients with rheumatoid arthritis. PLoS One 4: e6242. 19606218 10.1371/journal.pone.0006242 PMC2707000

[26] Wolfe F (2009) The many myths of erythrocyte sedimentation rate and C-reactive protein. J Rheumatol 36: 1568–69. 19671807 10.3899/jrheum.090386

[27] Dresselhaus TR, Peabody JW, Luck J, Bertenthal D (2004) An evaluation of vignettes for predicting variation in the quality of preventive care. J Gen Intern Med 19: 1013–8. 15482553 10.1007/s11606-004-0003-2 PMC1492573

[28] Peabody JW, Luck J, Glassman P, Dresselhaus TR, Lee M (2000) Comparison of vignettes, standardized patients, and chart abstraction: a prospective validation study of 3 methods for measuring quality. JAMA 283: 1715–22. 10755498 10.1001/jama.283.13.1715

[29] Peabody JW, Luck J, Glassman P, Jain S, Hansen J, et al (2004) Measuring the quality of physician practice by using clinical vignettes: a prospective validation study. Ann Intern Med 141: 771–80. 15545677 10.7326/0003-4819-141-10-200411160-00008

[30] Fox MG, Stephens T, Jarjour WN, Anderson MW, Kimpel DL (2012) Contrast-enhanced magnetic resonance imaging positively impacts the management of some patients with rheumatoid arthritis or suspected RA. J Clin Rheumatol. 18: 15–22. 10.1097/RHU.0b013e31823e5ab3 22157267

[31] Wennberg DE (1998) Variation in the delivery of health care: the stakes are high. Ann Intern Med 128: 866–8. 9599201 10.7326/0003-4819-128-10-199805150-00012

[32] Luce BR, Kramer JM, Goodman SN, Connor JT, Tunis S, et al (2009) Rethinking randomized clinical trials for comparative effectiveness research: the need for transformational change. Ann Intern Med 151: 206–9. 19567619 10.7326/0003-4819-151-3-200908040-00126

[33] Peabody JW, Liu A (2007) A cross-national comparison of the quality of clinical care using vignettes. Health Policy Plan 22: 294–302. 17660225 10.1093/heapol/czm020

[34] Solon O, Woo K, Quimbo SA, Shimkhada R, Florentino J, et al (2009) A novel method for measuring health care system performance: experience from QIDS in the Philippines. Health Policy Plan 24: 167–74. 19224955 10.1093/heapol/czp003 PMC2733796

